# Multi-Target Anti-Alzheimer Activities of Four Prenylated Compounds from *Psoralea Fructus*

**DOI:** 10.3390/molecules23030614

**Published:** 2018-03-08

**Authors:** Qing-Xia Xu, Ying Hu, Gui-Yang Li, Wei Xu, Ying-Tao Zhang, Xiu-Wei Yang

**Affiliations:** 1 State Key Laboratory of Natural and Biomimetic Drugs, Department of Natural Medicines, School of Pharmaceutical Sciences, Peking University Health Science Center, Peking University, No. 38, Xueyuan Road, Haidian District, Beijing 100191, China; xqx3081@163.com (Q.-X.X.); huying3916@163.com (Y.H.); high-xu@163.com (W.X.); 2 Department of Pharmacology, State Province Key Laboratories of Biomedicine and Pharmaceutics of China, Key Laboratory of Cardiovascular Research, Ministry of Education, College of Pharmacy, Harbin Medical University, No. 157, Baojian Road, Nangang District, Harbin 150086, China; gyli0612@outlook.com

**Keywords:** *Psoraleae Fructus*, Alzheimer’s, bavachin, bavachinin, bavachalcone, isobavachalcone, oxidative damage, neuroinflammation, BV-2 microglia, amyloid β-peptide 42

## Abstract

Alzheimer’s disease (AD) is an age-related neurodegenerative disease that is mediated by multiple signaling pathways. In recent years, the components of *Psoralea Fructus* (PF) have demonstrated some anti-Alzheimer effects both in vitro and in vivo. To further reveal the active compounds of PF and their mechanisms regulating key targets of AD, in this study, we identified four prenylated compounds from the 70% ethanolic aqueous extract of PF, namely bavachin, bavachinin, bavachalcone, and isobavachalcone. Multi-target bioactivity analysis showed that these compounds could differentially inhibit neuroinflammation, oxidative damage, and key AD-related protein targets, such as amyloid β-peptide 42, β-secretase, glycogen synthase kinase 3β, and acetylcholinesterase. These compounds may generate beneficial effects in AD prevention and treatment.

## 1. Introduction

The Leguminosae plant, *Cullen corylifolium* (L.) Medik. (syn. *Psoralea corylifolia* L.), is a medicinal plant widely distributed in China, India and Southeastern Asian countries. *Psoralea Fructus* (PF), the dried mature fruits (*Psoralea Fructus*, PF) of this plant, has been used as a traditional Chinese medicine for the treatment of erectile dysfunction, pollakiuria, nephritis, osteoporosis, and cerebral and cardiac vascular diseases, etc. [1], and is also a well-known health supplement ingredient [2]. PF was reported to contain flavonoids, coumarins, meroterpene phenols, and benzofurans with molecular diversity, and some of them exhibited estrogen-like, osteoblastic, anti-oxidant, anticancer, anti-inflammatory, antidepressant, hepatoprotective activities [3], as well as antibacterial effects [3,4].

Recent reports showed that *Psoralea Fructus* extract (PFE), and one or more of its active components, have inhibitory effects on neuroinflammation [5] and some key enzymes of the central nervous system (CNS), such as monoamine oxidases [6], acetylcholinesterase (AChE) [7], and β-secretase (BACE1) [8], which may partially account for their beneficial effects in Alzheimer’s disease (AD) [9] and Parkinson’s disease (PD) models [5,10]. As we have addressed in our previous reports, long-term dietary intake of the total prenylflavonoids (TPFB) of PF at 50 mg/kg·day significantly improved cognitive performance and AD-like neurobiochemical changes in an age-related AD mouse model SAMP8 [9], and some PF compounds could modulate amyloid β-peptide 42 (Aβ42) aggregation process in vitro [11]. In addition, we have also demonstrated that major compounds of PF could be absorbed and distributed to the cerebral nuclei of Sprague-Dawley rats after oral administration of a single dose of PFE at 1.2 g/kg [12]. These data suggested the potential value of PF compounds in AD prevention and treatment, thus motivated us to further explore the active components in PF. In this study we identified four structurally related compounds from PF, namely bavachin (**1**), bavachinin (**2**), bavachalcone (**3**), and isobavachalcone (**4**). We further examined their in vitro anti-AD activities involving multiple drug targets containing Aβ42, BACE1, glycogen synthase kinase 3β (GSK-3β), AChE, as well as neuroinflammation and oxidative damage.

## 2. Results and Discussion

### 2.1. Chemical Structures of the Purified Compounds

**Figure 1:**
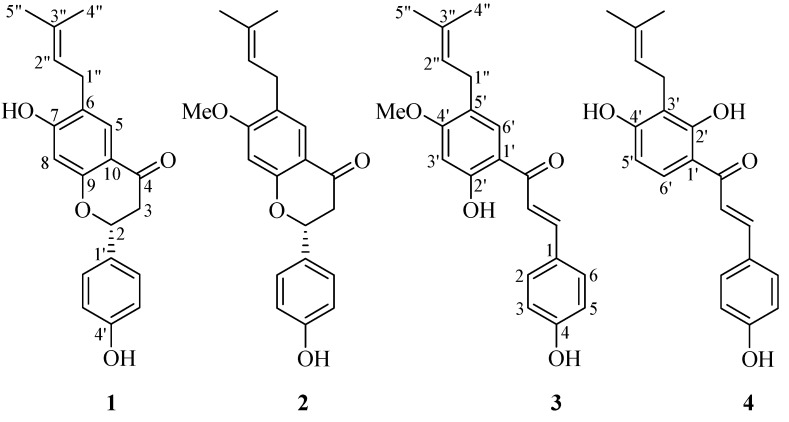
The structures of compounds **1**–**4** isolated from *Psoraleae Fructus*. (**1**) Bavachin, (**2**) Bavachinin, (**3**) Bavachalcone, (**4**) Isobavachalcone.

Silica gel column (SGC) chromatography was performed to yield four compounds from the ethyl acetate (EtOAc) extract of PF, they were identified as known prenylated compounds by comparison of their spectral data with those in the literatures: bavachin (**1**) [13], bavachinin (**2**) [14], bavachalcone (**3**) [15], and isobavachalcone (**4**) [16,17] (Figure 1). These compounds are structurally related molecules either with a skeleton of flavanone or chalcone, which may convert to each other by spontaneous or enzymic rearrangement.

### 2.2. Anti-Neuroinflammatory Effect in BV-2 Microglia

Although microglia represent about only 10% of the total cell population in the central nervous system (CNS) [18,19], they are the resident macrophage-like cells with a broad role in the brain’s innate immunity and in inflammatory neuropathologies [20], and have been demonstrated to have a critical role in the pathogenesis and progression of AD and PD, thus representing a potential target for therapeutic intervention [21]. Most work on microglial activation and signaling has been performed in vitro, frequently, by using cell lines, such as BV-2. BV-2, cells were derived from raf/myc-immortalised murine neonatal microglia and are the most frequently used substitute for primary microglia. These cells are highly responsive to toll-like receptor 4 (TLR4) agonists, such as lipopolysaccharide (LPS), which triggers the production and release of an array of neuroinflammatory mediators, such as nitric oxide (NO) and proinflammatory cytokines tumor necrosis factor (TNF)-α and interleukin (IL)-6. BV-2 cell lines or model have been extensively used for pharmacological and biological studies, and for many important immunological discoveries [22]. On the other hand, NO is a short-living free radical that is produced from *L*-arginine by constitutive nitric oxide synthase (cNOS) and inducible nitric oxide synthase (iNOS) within mammalian immune, cardiovascular, and neural systems, where it functions as a signaling or cytotoxic molecule. Low concentration of NO participates in neurotransmission and vasodilation, whereas the overproduction of NO was responsible for inflammation [23], therefore, NO inhibitor is believed to have therapeutic potential for the treatment of inflammation accompanying overproduction of NO [24].

**Figure 2:**
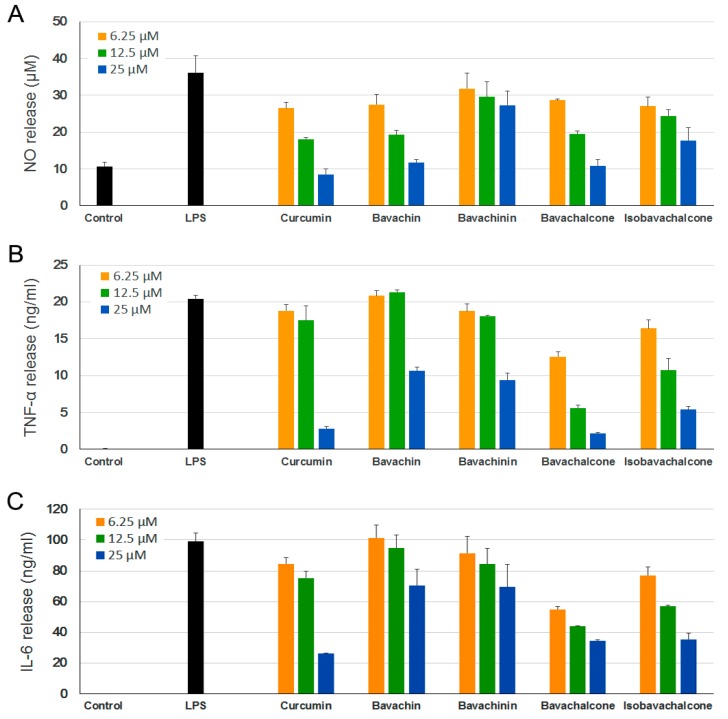
Anti-neuroinflammatory effects of *Psoralea Fructus* (PF) compounds. BV-2 cells were incubated with different concentrations of PF compounds for 1 h followed by treatment with 0.5 μg/mL lipopolysaccharide (LPS) for 24 h. The culture supernatant was aliquoted. (**A**) Nitric oxide (NO) levels were determined using Griess reaction; (**B**) Tumor necrosis factor (TNF)-α and (**C**) interleukin (IL)-6 levels were measured using ELISA. Curcumin was used as a positive control. Data are expressed as mean ± SEM.

Accordingly, we first examined the effect of the above-mentioned PF compounds (**1**–**4**) on LPS-induced NO production in BV-2 microglia cell model, in which curcumin was used as a positive control [25]. These compounds (1–25 µM) and LPS (0.1–1 µg/mL) were found to be non-toxic to BV-2 cells under tested concentrations according to MTT (3-(4,5-dimethyl-2-thiazolyl)-2,5-diphenyl-2*H*-tetrazolium bromide) assays [26] (data not shown). As shown in Figure 2A, LPS (0.5 µg/mL) stimulation strongly increased NO release of BV-2 cells compared with untreated controls, and pretreatment with all four PF compounds significantly inhibited LPS-induced NO production in dose-dependent manners. Bavachin and bavachalcone demonstrated strong inhibition on NO production, which were comparable to curcumin.

Notably, NO inhibition activities of bavachalcone and bavachin exhibited dose-dependent inhibitory effects with IC_50_ values of 6.10 and 7.71 µM, respectively, which were not statistically different from that of curcumin (IC_50_ = 6.61 µM). Bavachinin and isobavachalcone exhibited moderate inhibitory effects with IC_50_ values of 27.06 and 19.32 µM, respectively, which were weaker than curcumin (*p* < 0.005).

LPS-activated microglia extensively induce the production of proinflammatory cytokines, IL-6 and TNF-α [27], which leads to neuroinflammation. Plenty of studies have suggested chronic neuroinflammation as a key factor in the development of various neurodegenerative diseases, including AD, PD, and amyotrophic lateral sclerosis [28,29]. Thus, substances that suppress the release of these proinflammatory cytokines could be valuable for the treatment of neuroinflammatory diseases. We further examined the effect of PF compounds on LPS-induced production of inflammatory cytokines TNF-α and IL-6. Similarly, all of the PF compounds demonstrated dose-dependent inhibition on release of both cytokines (Figure 2B,C), and bavachalcone proved to be the strongest inhibitor, which was comparable to curcumin as well.

### 2.3. Anti-Oxidative Effects in PC-12 Cells

**Figure 3:**
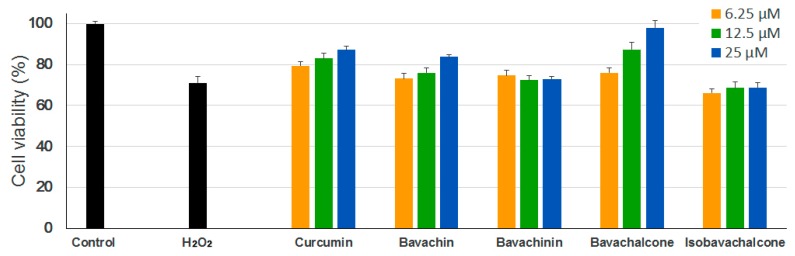
Anti-oxidative effects of PF compounds. PC-12 cells were incubated with different concentrations of PF compounds for 24 h followed by treatment with 450 µM of H_2_O_2_ solution for 2 h. Cell viability was measured using MTT assay. Curcumin was used as a positive control. Data are expressed as mean ± SEM.

In neurodegenerative diseases, H_2_O_2_ is one of the most important mediators of oxidative stress that is detected under pathological conditions. H_2_O_2_ generation is required to mediate the complete sequence of events occurring in oxidative stress-induced neuronal cell death. It has been suggested that oxidative stress may play an important role in the development of AD, which involves the overproduction of reactive oxygen species (ROS) and the accumulation of oxidative damage [30,31,32]. To assess the anti-oxidative effects of the PF compounds, we used H_2_O_2_-induced oxidative damage model in PC-12 cells (rat pheochromocytoma). As shown in Figure 3, H_2_O_2_ treatment significantly reduced the viability of PC-12 cells as compared with untreated controls, however, only bavachin and bavachalcone exerted significant protective effects against H_2_O_2_-induced neuronal cell damage under examined concentrations, which is comparable or superior to curcumin.

### 2.4. Inhibitory Effects on BACE-1, GSK-3β, and AChE

**Figure 4:**
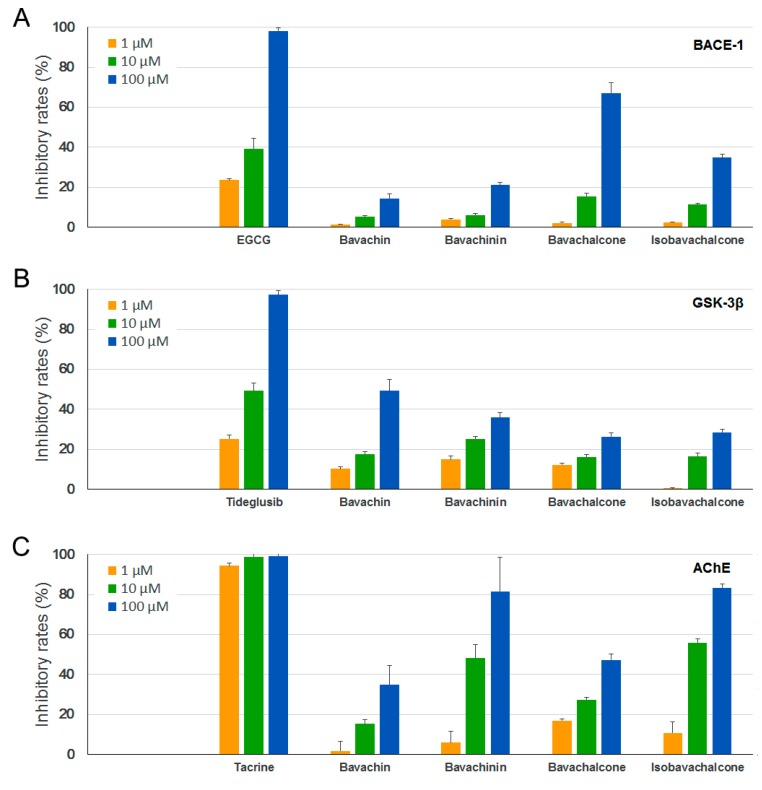
Inhibitory effects of PF compounds on (**A**) β-secretase (BACE-1); (**B**) glycogen synthase kinase 3β (GSK-3β); and (**C**) acetylcholinesterase (AChE). Epigallocatechin gallate (EGCG), tideglusib, and tacrine were used as positive controls, respectively. Data are expressed as mean ± SEM.

Several key enzymes have been implicated in major hypotheses of AD origin and are thus considered as potential drug targets for AD treatment [33,34,35]. BACE-1 is a membrane-associated aspartic protease that cleaves amyloid precursor protein (APP), and with the activity of γ-secretase, produces a 40- or 42-residue neurotoxic peptide called amyloid-β (Aβ). GSK-3β is a multi-functional protein kinase that is involved in various cellular processes, and also acts as a critical factor in AD development through phosphorylating the microtubule-associated protein Tau and promoting neuroinflammation. AChE is the primary cholinesterase that breaks down the neurotransmitter acetylcholine at the synaptic cleft, which progressively decreases in AD brains, resulting in learning and memory deficits. To evaluate the activities of PF compounds on these drug targets, we carried out in vitro enzyme assays using recombinant human BACE-1, GSK-3β, and AChE. However, only moderate to weak inhibitory activities were observed for the four PF compounds on these enzymes (Figure 4A–C).

### 2.5. Inhibitory Effects on Aggregation of Aβ42

**Figure 5:**
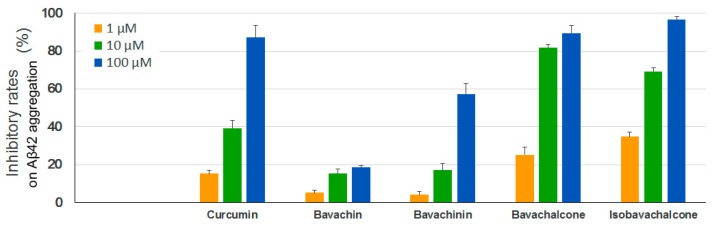
Inhibitory effects of PF compounds on Aβ42 aggregation. Synthetic Aβ42 peptide was diluted in PBS to 50 μM, incubated with different concentrations of PF compounds at 37 °C for 24 h, mixed with ThT solutions and measured at 440/490 nm (excitation/emission). Curcumin was used as a positive control. Data are expressed as mean ± SEM.

A large number of studies have demonstrated that the aggregation of the amyloid-β peptide, especially Aβ42, leads to toxic oligomers and amyloid fibrils linking to the development of AD [33,34,35]. We thus performed a Thioflavin T (ThT) fluorescence assay to test the anti-aggregation effects of the four PF compounds using synthetic human Aβ42. As shown in Figure 5, both bavachalcone and isobavachalcone exhibited strong inhibitory activitis on Aβ42 aggregation, which were comparable to the positive control curcumin, whereas the potency of bavachinin was lower than that of curcumin and bavachin showed only minor inhibitory effects.

Previously we have demonstrated that the commercial chemical standard isobavachalcone and bavachinin inhibited Aβ42 aggregation through different mechanisms, and the inhibition potency of bavachinin was relatively lower than that of isobavachalcone [11], which is consistent with the present findings. However, whether these compounds could directly bind to Aβ42 remains unknown.

**Figure 6:**
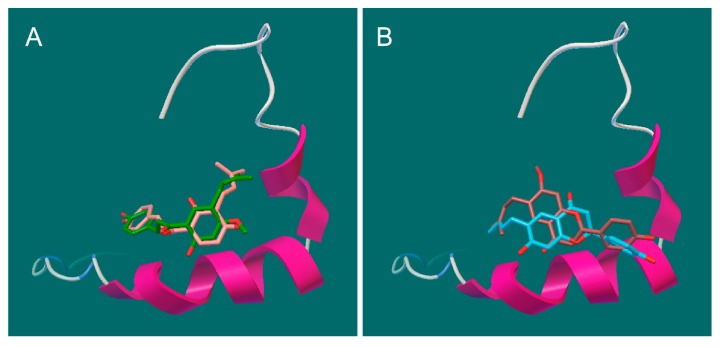

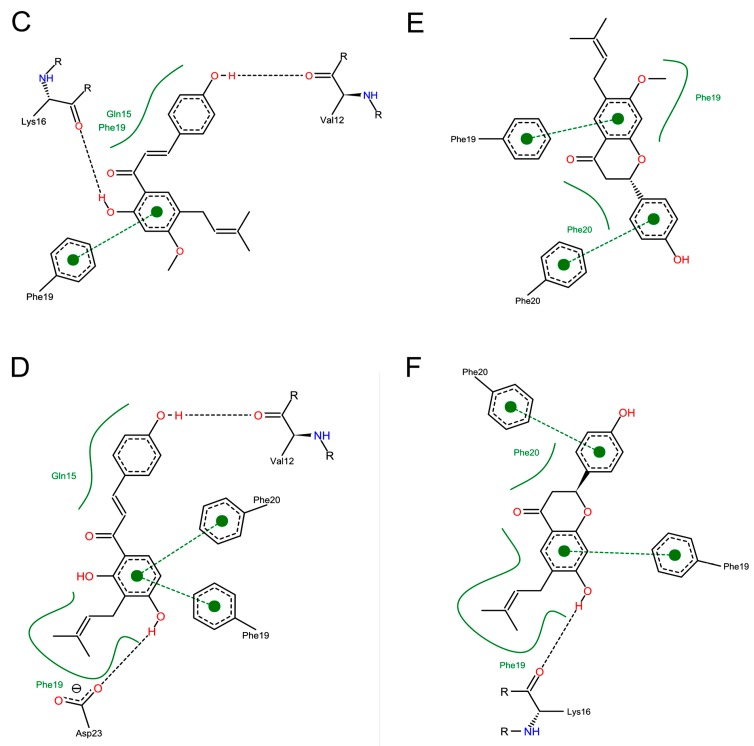
Docking studies of PF compounds and Aβ42 monomer. The PF molecules were docked into the whole surface of Aβ42 monomer (1Z0Q) using Autodock Vina. The best docking poses were viewed using Pymol and PoseView. The binding modes are shown for (**A**) bavachalcone & isobavachalcone and (**B**) bavachin & bavachinin, binding at the central hydrophobic cluster K^16^LVFF^20^; The possible interactions are shown for (**C**) bavachacone; (**D**) isobavachalcone; (**E**) bavachinin; and (**F**) bavachin.

To further understand the possible interactions between Aβ42 and the PF compounds, we performed molecular docking analysis using Autodock Vina [36,37]. Previous studies suggest that the K^16^LVFF^20^ segment in the Aβ sequence is crucial for the peptide’s oligomeric properties as well as fibrillogenetic behavior [38,39,40]. The three-dimensional (3D) structure 1Z0Q of Aβ42 in aqueous solution contains 30 conformers and demonstrates a quite stable helical structure of this central hydrophobic cluster comparing to much more variable terminal regions. Not surprisingly, all the best docking poses of the PF compounds were found to bind at this region (Figure 6A,B). The key interactions (Figure 6C–F) include hydrophobic contacts (Gln15, Phe19 and Phe 20), π-π stacking (Phe19 and Phe 20), and hydrogen bonds (Val12, Lys16, Asp23). The different binding modes of PF compounds could be primarily linked to their different backbones. The chalcone backbone of bavachalcone and isobavachalcone is more flexsible, which allows for them to fit more easily to the conformations of Aβ42 and enables more hydrogen bonds than the flavanone of bavachin and bavachinin. These results indicated that PF compounds might inhibit aggregation process of Aβ42 through direct binding to its amyloidogenic region. Bavachalcone and isobavachalcone may stablilize Aβ42 monomers through their strong bindings, whereas bavachinin might induce intricate conformational changes of Aβ42 through the binding, which leads to the off-pathway aggregation [11].

## 3. Experimental Section

### 3.1. General Information

Open column chromatography separation was carried out using silica gel (200–300 mesh; Qingdao Marine Chemical Co., Qingdao, China). Thin layer chromatography was conducted on silica gel GF_254_ plates (Merck, Darmstadt, Germany). ^1^H (400 MHz) and ^13^C (100 MHz) were run on a Bruker AV 400 spectrometer (Bruker BioSpin AG Facilities, Fällanden, Switzerland). Electron impact mass spectra (EI-MS) data were obtained with a Thermo Finnigan Trace 2000 GC-MS spectrometer (Thermo Finnigan Inc., San Jose, CA, USA). Sanyo MCO-15 AC carbon dioxide (CO_2_) incubator (Sanyo Electric Co., Ltd., Osaka, Japan).

### 3.2. Plant Material

The mature fruits of *Psoralea corylifolia* L. were collected from the Gengma county in Lincang city of the Yunnan province of China in October 2016 and were identified by Prof. Xiu-Wei Yang of the School of Pharmaceutical Sciences, Peking University Health Science Center, Peking University. A voucher specimen (No. BGZ201610) was deposited in State Key Laboratory of Natural and Biomimetic Drugs (Peking University, Beijing, China).

### 3.3. Chemicals and Reagents

Sodium dodecyl sulfate, isopropanol, hydrochloric acid, hydrogen peroxide solution (H_2_O_2_, 3 wt. % in H_2_O), MTT, LPS, Griess reagent, dimethylsulfoxide (DMSO), and curcumin were obtained from Sigma-Aldrich (St. Louis, MO, USA). Dulbecco’s modified Eagle’s medium (DMEM), fetal bovine serum (FBS), trypsin, penicillin-streptomycin solution, and phosphate buffered saline were obtained from Gibco^®^ (Life Technologies Inc., Grand Island, NY, USA). 96-Well plates and cell culture flasks were obtained from Corning Inc. (Cambridge, MA, USA). Hank’s Balanced Salts Solution (HBSS) and other chemicals, methanol (MeOH), EtOAc, ethanol (EtOH), *n*-butanol (BuOH), *n*-hexane, and petroleum ether, were of analytical grade from Beijing Chemical Works (Beijing, China). Milli-Q water (H_2_O) (Millipore, Bedford, MA, USA) was used throughout the study.

### 3.4. Isolation of Compounds from the Fruits of P. corylifolia

The dried mature fruits powder (47.9 kg) was extracted with 70% aqueous EtOH (479 kg for 2 h first and 384 kg for 2 h each time for the second and third extraction) under reflux. The extracts were combined and then concentrated under reduced pressure to afford a residue (8.2 kg, yield 17.12%). The residue (6.0 kg) was suspended in H_2_O and extracted in *n*-hexane (8 L × 8), EtOAc (8 L × 8) and BuOH (8 L × 8), successively, to give corresponded extract for 1.2 kg, 2.2 kg, and 0.7 kg. The EtOAc extract (2.0 kg) was subjected to silica gel column (SGC) (200 mm i.d. × 850 mm) to yield 19 fractions (Fr. A–S). The Fr. C (500 g) was subjected to SGC (140 mm i.d. × 800 mm) to afford 9 subfractions (Fr. C-1–C-9). The Fr. C-1 was recrystallized from MeOH to give compound **2** (40 g). The Fr. C-2 was recrystallized from MeOH to afford compound **3** (1 g) and the mother liquor. The mother liquor was concentrated under reduced pressure and then subjected to SGC (55 mm i.d. × 700 mm) to yield crude compound **4**, which was recrystallized from petroleum ether-EtOAc (4:1) to give pure compound **4** (2.5 g). The Fr. C-4 was recrystallized from MeOH to obtain compound **1** (0.87 g).

### 3.5. Compound Characterization

*Bavachin* (**1**): Needles (MeOH). EI-MS *m*/*z* 324 [M]^+^. ^1^H-NMR (400 MHz, DMSO-*d*_6_) δ: 5.39 (1H, dd, *J* = 13.0, 2.7 Hz, H-2), 3.07 (1H, dd, *J* = 16.7, 13.0 Hz, H_α_-3), 2.62 (1H, dd, *J* = 16.7, 2.7 Hz, H_β_-3), 7.47 (1H, s, H-5), 6.40 (1H, s, H-8), 7.32 (2H, d, *J* = 8.4 Hz, H-2’, 6’), 6.81 (2H, d, *J* = 8.4 Hz, H-3’, 5’), 3.18 (2H, br d, *J* = 7.2 Hz, H-1”), 5.27 (1H, tt, *J* = 7.2, 1.3 Hz, H-2”), 1.71 (3H, s, H-4”), 1.66 (3H, s, H-5”), 9.57 (1H, br s, 4’-OH), 10.61 (1H, br s, 7-OH); ^13^C-NMR (100 MHz, DMSO-*d*_6_) *δ*: 79.2 (C-2), 43.5 (C-3), 190.4 (C-4), 122.5 (C-5), 122.9 (C-6), 157.8 (C-7), 102.3 (C-8), 161.6 (C-9), 113.3 (C-10), 132.1 (C-1’), 128.4 (C-2’, 6’), 115.4 (C-3’, 5’), 162.7 (C-4’), 27.4 (C-1”), 127.0 (C-2”), 129.7 (C-3”), 25.8 (C-4”), 17.8 (C-5”).

*Bavachinin* (**2**): Needles (MeOH). EI-MS *m*/*z* 338 [M]^+^. ^1^H-NMR (400 MHz, CDCl_3_) *δ*: 5.37 (1H, dd, *J* = 13.3, 2.7 Hz, H-2), 3.05 (1H, dd, *J* = 16.9, 13.3 Hz, H_α_-3), 2.79 (1H, dd, *J* = 16.9, 2.7 Hz, H_β_-3), 7.69 (1H, s, H-5), 6.44 (1H, s, H-8), 3.84 (3H, s, 7-OMe), 7.32 (2H, d, *J* = 8.5 Hz, H-2’, 6’), 6.92 (2H, d, *J* = 8.5 Hz, H-3’, 5’), 3.24 (2H, dd, *J* = 7.2, 1.3 Hz, H-1”), 5.27 (1H, tt, *J* = 7.5, 1.3 Hz, H-2”), 1.73 (3H, s, H-4”), 1.69 (3H, s, H-5”); ^13^C-NMR (100 MHz, CDCl_3_) *δ*: 79.8 (C-2), 43.9 (C-3), 192.0 (C-4), 127.1 (C-5), 125.0 (C-6), 164.4 (C-7), 98.8 (C-8), 162.6 (C-9), 113.7 (C-10), 55.7 (7-OMe), 130.5 (C-1’), 127.9 (C-2’, 6’), 115.7 (C-3’, 5’), 156.5 (C-4’), 27.7 (C-1”), 121.6 (C-2”), 133.1 (C-3”), 25.8 (C-4”), 17.7 (C-5”).

Bavachalcone (**3**): Square crystal (MeOH). EI-MS m/z 338 [M]^+^. ^1^H-NMR (400 MHz, CDCl_3_) δ: 7.43 (1H, d, J = 15:4 Hz, H-α), 7.82 (1H, d, J = 15:4 Hz, H-β), 7.54 (2H, dd, J = 8.5, 1.9 Hz, H-2, 6), 6.90 (2H, dd, J = 8.5, 1.9 Hz, H-3, 5), 6.44 (1H, s, H-3’), 7.59 (1H, s, H-6’), 3.26 (2H, dd, J = 7.2, 1.3 Hz, H-1”), 5.28 (1H, tt, J = 7.2, 1.3 Hz, H-2”), 1.77 (3H, s, H-4”), 1.74 (3H, s, H-5”), 3.87 (3H, s, 4’-OMe), 13.58 (1H, br s, 2’-OH); ^13^C-NMR (100 MHz, CDCl_3_) δ: 191.9 (C=O), 118.0 (C-α), 144.1 (C-β), 127.7 (C-1), 130.6 (C-2, 6), 116.0 (C-3, 5), 158.1 (C-4), 113.3 (C-1’), 164.2 (C-2’), 99.3 (C-3’), 165.2 (C-4’), 121.8 (C-5’), 129.7 (C-6’), 28.0 (C-1”), 122.3 (C-2”), 133.0 (C-3”), 17.8 (C-4”), 25.8 (C-5”), 55.7 (4’-OMe).

*Isobavachalcone* (**4**): Yellowish amorphous powder (petroleum ether-EtOAc/4:1). EI-MS *m*/*z* 324 [M]^+^*.* ^1^H-NMR (400 MHz, CD_3_OD) *δ*: 7.54 (1H, d, *J* = 15:4 Hz, H-α), 7.71 (1H, d, *J* = 15:4 Hz, H-β), 7.53 (2H, br d, *J* = 8.6 Hz, H-2, 6), 6.78 (2H, br d, *J* = 8.6 Hz, H-3, 5), 6.37 (1H, d, *J* = 9.0 Hz, H-5’), 7.75 (1H, d, *J* = 9.0 Hz, H-6’), 3.27 (2H, dd, *J* = 7.2, 1.3 Hz, H-1”), 5.18 (1H, tt, *J* = 7.2, 1.3 Hz, H-2”), 1.71 (3H, s, H-4”), 1.60 (3H, s, H-5”); ^13^C-NMR (100 MHz, CD_3_OD) *δ*: 193.7 (C=O), 118.6 (C-α), 145.3(C-β), 127.9 (C-1), 131.7 (C-2, 6), 116.9 (C-3, 5), 161.4(C-4), 114.5 (C-1’), 163.6 (C-2’), 116.6 (C-3’), 165.1 ( C-4’), 108.2 (C-5’), 130.4 (C-6’), 22.5 (C-1”), 123.6 (C-2”), 131.8 (C-3”), 26.0 (C-4”), 17.9 (C-5”).

### 3.6. Anti-Neuroinflammatory Effect in LPS-induced BV-2 Microglia

#### 3.6.1. Inhibition Assay on NO Release

BV-2 microglial cell line was purchased from the cell bank of Peking Union Medical College (PUMC, Beijing, China). Cells were maintained in DMEM containing 10% FBS, in a constant humidity atmosphere of 5% CO_2_ and 95% air at 37 °C. For NO inhibition assay, BV-2 cells were seeded in 96-well culture plates at a density of 2 × 10^4^ cells/well for 24 h, and then stimulated with LPS (0.5 μg/mL) and treated with various concentrations of assay compounds for 24 h. After that, the cell culture supernatant (100 μL) was collected to react with Griess reagent [41] (100 μL) for 15 min at room temperature. The nitrite in culture medium was measured as an indicator of NO production. NaNO_2_ was used to generate a standard curve, and NO production was determined by measuring the optical density at 540 nm by a Multiskan MK3 Automated Microplate Reader (Thermo-Labsystems, Franklin, MA, USA). The experiments were performed in parallel for three times, and curcumin was used as a positive control. Cell viability (>95%) was assessed using an MTT assay. The IC_50_ value of each compound is defined as the concentration (μM) that caused 50% inhibition of NO production. The IC_50_ values were calculated by the software SPSS 16.0 (SPSS Inc., Chicago, IL, USA).

#### 3.6.2. Inhibition Assay on Cytokine Release

BV2 cells were treated with or without compounds **1**–**4** at the assayed concentrations (6.25–25.00 µM) for 1 h, and then stimulated with LPS for 24 h. The culture media were collected to determine the levels of IL-6 and TNF-α present in each sample using commercially available enzyme-linked immunosorbent assay (ELISA) kits (Multi Sciences Biotech, Co., Ltd., Hangzhou, China). The assays were performed according to the manufacturer’s instructions.

### 3.7. Anti-Oxidative Effect in H_2_O_2_-Induced PC-12 Cells

PC-12 cells were maintained as BV-2 cells as described above. MTT assay was performed for cell toxicity analysis of the compounds. For anti-oxidative assay, PC-12 cells were seeded into 96-well culture plate for 24 h and incubated with different concentrations of compounds for 24 h followed by treatment with 450 µM of H_2_O_2_ solution for 2 h. Curcumin was used as a positive control. The cell viability was measured using MTT assay to evaluate the oxidative damage of the cells and the protective effects of the compounds.

### 3.8. Enzyme Inhibition Assays of BACE-1, GSK-3β and AChE

BACE-1 and AChE activity assays were performed using assay kits from Sigma-Aldrich (St Louis, MO, USA). GSK-3β activity assays were conducted using recombinant human GSK-3β and ADP-Glo™ kinase assay kit from Promega (Madison, WI, USA). All of the assays were carried out according to the manufacturer’s instructions. Epigallocatechin gallate (EGCG), tacrine, and tideglusib were used as positive controls, respectively.

### 3.9. Anti-Aggregation Assay of Aβ42

Synthetic Aβ42 peptide (ChinaPeptides, Shanghai, China) was prepared and diluted in PBS (pH 7.4) to 50 μM, as described previously [11]. The peptide was incubated at 37 °C in the presence or absence of test compounds for 24 h without agitation. Curcumin was used as a positive control. The incubated samples (10 μL) were mixed with 40 μL ThT solutions (25 μM in 0.2 M Glycine-NaOH buffer, pH 8.5), placed in the dark for 10 min, and then measured at 440/490 nm (excitation/emission) in a fluorescence spectrophotometer (F96Pro, Shanghai Lengguang Technology Co., Ltd., Shanghai, China).

### 3.10. Docking Studies of Aβ42 Monomer

All the 3D structures of the ligands were downloaded from the website of PubChem (pubchem.ncbi.nlm.nih.gov). The 3D NMR structure of Aβ42 monomer (PDB ID: 1Z0Q) were downloaded from the website of Protein Data Bank (PDB; www.rcsb.org). Docking studies were conducted using Autodock Vina [36,37]. The protein structures were prepared and imported with the default parameters. The ligands were docked into the whole surface of Aβ42 monomer. The best poses of the ligands and their key interactions were viewed using Pymol [37] and PoseView (poseview.zbh.uni-hamburg.de).

### 3.11. Statistical Analysis

Data were analyzed by SPSS statistics package v.20.0 (SPSS Inc., Chicago, IL, USA). Statistical significances were calculated by Student′s *t*-test, and the differences were considered significant at *p* < 0.05. Results were expressed as the mean ± SEM.

## 4. Conclusions

Taken together, the four structurally related PF compounds that were identified in this study demonstrated multi-target anti-AD activities in vitro. Bavachalcone was found to be the most potent inhibitor which exerted comparable effects as curcumin, the well-known neuroprotective agent, on LPS-induced neuroinflammation, H_2_O_2_-induced neurodamage, and spontaneous Aβ42 aggregation. These new findings provided useful information for predicting anti-AD effective constituents of PF and their potential clinical values in the treatment of AD. The active ingredient bavachalcone has the potential to be developed into a drug and further investigation in vivo is needed.
